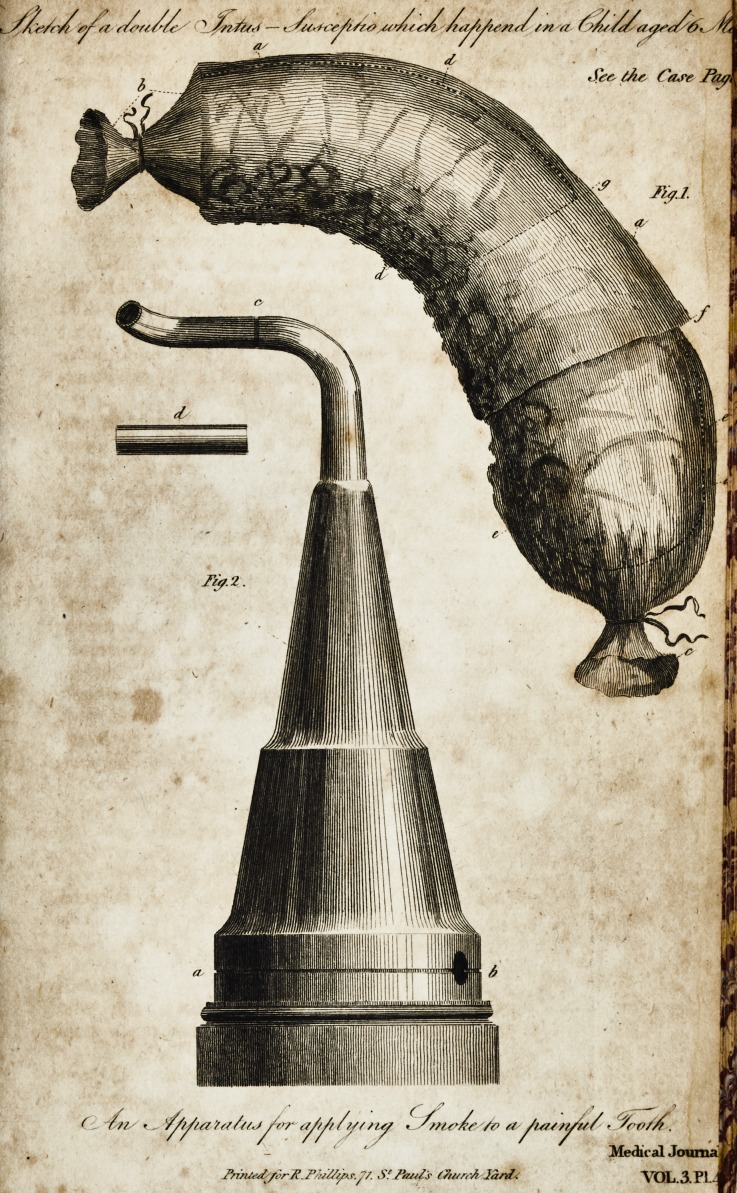# Observations on Odontalgia, with Dr. Brown's Explanation of an Apparatus That Has Been Employed in Relieving It
*Vide Plate in Medical Journal, Numb. XI.


**Published:** 1800-05

**Authors:** Joseph Brown

**Affiliations:** Terrace, Lower Street, Islington


*April* 18, 1800.

---

### *Observations on Odontalgia, with* Dr. Brown's *Explanation of an Apparatus* that has been employed in relieving it.*

---

To the Editors of the Medical and Physical Journal.

Gentlemen,

ODONTALGIA, or the Tooth-Ach, the moſt frequent and painful diſeaſe incident to the human body, is often occaſioned by being expoſed to a partial current of cold air, or to ſudden heat and cold; by reſiding in a marſhy or damp ſituation; by morbid matter within, by acrid matter attached to the

---

* Vide Plate in Medical Journal, Numb. XI.

the external ſurface of the teeth, and frequently by the muſcles and membranes of the jaw being affected by the ſame diatheſis which produces irritation, gout, and rheumatiſm in other parts. Women are more liable than men to rheumatiſms and to tooth-ach; they are moſt ſubject to both in pregnancy and during obſtructions of the menſtrual diſcharge.

It has alſo been obſerved, that mercurial preparations thrown into the ſyſtem, ſo as to affect the ſalival glands; nervous affections; ſipping hot liquors; frequent picking of the teeth with hard inſtruments; a ſcorbutic and ſome other acrimonious ſtate of the fluids, probably often occaſion a caries of the teeth; and I have known ſeveral families, who ſeemed particularly liable to their premature loſs and decay.

Having myſelf, during five years of my life, been harraſſed by frequent attacks of the tooth-ach, and the *dolor faciei*, a tormenting diſeaſe, which is noticed in p. 465 of the ſecond vol. of your excellent Medical Miſcellany, I read with the moſt lively intereſt, Dr. Dyce's communication "of an old and popular remedy for the tooth-ach,"* which is, doubtleſs, the ſame as the Doctor mentions (p. 270) as appearing "to many ſo extraordinary, having been practiſed only lately" at Aberdeen. The common people of that city, it ſeems, have an idea that the cauſe of the tooth-ach "is a worm, or worms, being engendered in the tooth affected." Juſt ſo, in former times, it was generally ſuppoſed, that worms contained in the cavity of the affected tooth, by irritating its nerve, occaſioned the acute pain that is ſometimes productive of watchfulneſs, fever, loſs of appetite, proſtration of ſtrength, (convulſions in children) and delirium, in perſons who have laboured under this exquiſitely painful diſeaſe.

Hence in *Regimen Sanitatis Salerni*,† p. 154:

"*Sic dentes ſerve porrorum collige grana,*

"*Ne carcas jure cum inſquiarno ſimul ure,*

"*Siſq; per embotum Fumum cape dente remotum.*"

"To cure the tooth-ach, take the ſeed of leeks,

When that fell pain annoyes and ſwells the cheeks;

But *seed* of HENBANE muſt be mixt among,

And burn them both, to make the ſmoke be ſtrong;

Hold thy mouth o'er, and ſo receive the fume,

The pain it ſlakes, and worms in teeth conſume,

If through a tunnil, you the ſmoke aſſume."

Sir

* Vide Medical Journal, p. 269.

† The SCHOOLE OF SALERNES, or Regiment of Health, dedicated unto the late High and Mighty King of England, from that Univerſity, and publiſhed, by conſent of learned phyſicians, for general good. LONDON, 1649.

Sir Hans Sloane gives an inſtance of the virtues of *ſemen hyoſciami*, in alleviating the tooth-ach. A perſon of quality, tormented with this racking pain, had an empiric recommended to him, who conveyed the ſmoke of burning ſeeds of henbane, by means of a funnel, into the hollow tooth, and thereby removed the pain; but, at the ſame time, there dropped ſome maggots from the tooth (as the operator pretended) into a pail of water placed underneath for that purpoſe. Sir Hans procured one of theſe maggots, which he ſent wrapt up to M. Lewenhoeck, at Delft, in Holland, where it arrived ſafe and alive. Upon examination, M. Lewenhoeck found it to be entirely like thoſe bred in ordinary rotten cheeſe; wherefore he procured ſome of theſe latter, and carefully fed them both, and that one Sir Hans Sloane had ſent, on the ſame cheeſe; and they were all, according to the uſual methods of nature, turned into ſmall ſcarabei, both being returned ſuch to Sir Hans from Holland. Upon the whole, though the henbane ſeeds cured the tooch-ach, it is highly probable, that the maggots had been conveyed thither, and let drop into the water by ſome ſlight of hand.

We learn from the writings of Dioſcorides and others, that *hyoſcyami*, which is a powerful remedy of the narcotic tribe, has been employed as an anodyne by the moſt illuſtrious phyſicians, from the earlieſt periods of medical hiſtory. It appears to have been the favourite ſedative of Celſus; he gave it internally to mitigate pain and procure ſleep; he uſed it externally as a collyrium, in caſes of opthalmia; and he employed it topically to aſſuage the pains of the teeth and gums. Accordingly, under the head of "*Dentium doloris varia remedia*," he ſays, with his uſual eloquence and preciſion, "The tooth-ach is a diſorder that may be juſtly ranked among the greateſt torments;" and he tells us, "if the pain be ſevere, a clyſter is uſeful, with hot cataplaſms applied to the cheek, as alſo ſome medicinal hot liquor held in the mouth, and frequently changed. For which purpoſe is uſed, henbane root, either in vinegar and water or diluted wine, with the addition of a little ſalt to either of them, and poppy heads not over dry, and mandrake roots prepared in the ſame manner; but in theſe three, care muſt be taken not to ſwallow what is in the mouth."

Mynſicht, who has long been reſpectfully named in our Diſpenſatories,* gives, in his *Medico-Chymicum*, the following formula, which are all deſigned for the uſe of perſons afflicted with painful affections of the teeth and gums, viz. Tinctura

* Vide his Tinctura Martis and Elixir Vitrioli, ſo highly commended by Fuller in his Medicina Gymnaſtica.

Odontalgia,* Pilulæ Odontalgicæ, Trochifci de Alumine cum radix Pyrethrum, Spiritus Odontalgicus; in all which he directs a certain portion of the feeds of henbane. Salmon, whofe labours feem not to have been duly appreciated by his countrymen, and Etmuller, prefcribe them for the fame purpofe. Boerhaave had his fenfes difordered by only making a plafter from this plant. Yet, as the various medicines I had hitherto employed were of little avail, and as I had fome teeth extracted, and obtained in one inftance only twelve hours refpite from my fufferings, the pain flying either to another tooth, or attacking the *Antrum maxillare*, I tried the effect of fumigating with

Sem. Hyofcy. p. ij. Carui. Cumin. aa. p. j. M.

After repeating the operation three times, I remained near a fortnight exempt from pain; I then had occafion to have recourfe to the fumigation again, and was again relieved, and in the mean time a permanent cure was obtained by the ufe of internal medicines, which had never occurred either to myfelf or any of the many fkilful and experienced practitioners who had been confulted, on what appeared to fome of them, as a cafe equally inveterate and extraordinary.

In cafe of a carious tooth, extraction is often the only radical remedy; but as in fome cafes extraction is improper, and as in many inftances it is obftinately avoided; with a view to relieve my friends labouring under pains of the teeth, I had a veffel made of copper, from which you have furnifhed the readers of your eftimable Journal,† with an elegant engraving.—The apparatus takes in pieces at the line *a. b.* and alfo at *c.* The bottom is fitted into a wooden ftand turned with a groove, to prevent its flipping out of the hand of the operator. In the centre of the bottom of this veffel, (as the tube at the bottom of a lanthorn for the reception of a candle) is a piece of metal fhaped to receive the bowl of a *large* tobacco-pipe, with an opening large enough to receive the ftalk of the pipe oppofite to the hole at *b.* The apparatus being taken afunder at *a. b,* the cup or bowl of an iron or common clay tobacco-pipe is made red hot, filled quickly with the feeds, placed in the aperture formed for its reception, the conical or upper part being reftored to its place, as in the engraving; the tube *c.* is turned upwards or downwards, as the pain may happen to be feated in the upper or lower jaw; ‡ and being placed in contact with

* That is inferted in Dr. Lewis's Difpenfatory, p. 330. 5th edit.

† See the plate in No. XI.

‡ The fmall tube marked *d,* is intended to be ufed when the pain is feated in the front teeth.

Sketch of a double Intus—Susceptio which happend in a Child aged 6 M

See the Case Pag

An Apparatus for applying Smoke to a painful Tooth.

Printed for R. Phillips. 71. St. Paul's Church Yard.

with it, the operator, applying his mouth to the tube of the pipe, forces by his breath, into the mouth of his patient, the smoke of the burning seeds, which, by their heating, sedative, and sialagogue effect, (sometimes it being necessary to repeat the process) generally afford the patient considerable relief. This method I practised for the ease of my friend, whenever I was required, till experience suggested to me more convenient and, perhaps, more effectual methods of affording at least temporary relief to those who are suffering by one of the most frequent and tormenting maladies.

With the best wishes for the success of your laudable labours, I have the honour to be,

GENTLEMEN,

Your most obedient servant,

JOSEPH BROWN.

*Terrace, Lower Street,*

*Islington, April* 18, 1800.

## Figures and Tables

**Fig. 2. f1:**